# Comprehensive genomic and metabolomic analysis revealed the physiological characteristics and pickle like odor compounds metabolic pathways of *Bacillus amyloliquefaciens* ZZ7 isolated from fermented grains of Maotai-flavor baijiu

**DOI:** 10.3389/fmicb.2023.1295393

**Published:** 2023-10-30

**Authors:** Liang Yang, Shuangran Zeng, Meidi Zhou, Yuetao Li, Zeyuan Jiang, Pingyan Cheng, Chunlin Zhang

**Affiliations:** ^1^Department of Brewing Engineering, Moutai Institute, Renhuai, China; ^2^College of Life Sciences, Shihezi University, Shihezi, China; ^3^Guizhou Xijiu Co., Ltd., Renhuai, China

**Keywords:** Maotai flavor baijiu, pickle like odor, *Bacillus amyloliquefaciens* ZZ7, metabolomics, whole genome sequencing and annotation, sulfur compounds

## Abstract

Pickle like odor (PLO) is one of the main defective flavors of Maotai flavor baijiu (MFB). Understanding and controlling the PLO compounds producing strains not only solves the problem of PLO from the source, but also ensures the high-quality production of MFB. However, the relevant research on PLO compounds producing strains has not been reported in MFB. In this study, we identified a *Bacillus amyloliquefaciens* ZZ7 with high yield of PLO compounds in the fermented grains of MFB, and measured its physiological characteristics. It produces 627 volatile compounds and 1,507 non-volatile compounds. There are 7 volatile sulfur compounds that cause the PLO, the content of dimethyl disulfide, dimethyl trisulfide, and dimethyl sulfur is relatively high, accounting for 89.43% of the total volatile sulfur compounds. The genome size of *B. amyloliquefaciens* ZZ7 is 3,902,720 bp with a GC content of 46.09%, and a total of 3,948 protein coding genes were predicted. Moreover, the functional annotation of coding genes and an assessment of the metabolic pathways were performed by genome annotation, showing it has strong ability to transport and metabolize amino acids and carbohydrates. Comprehensive genomic and metabolomic analysis, the metabolic pathway of PLO compounds of *B. amyloliquefaciens* ZZ7 was revealed, which mainly involves 12 enzymes including sulfate adenylyltransferase, cysteine synthase, cystathionine γ-synthase, etc. This work provides biological information support at both genetic and metabolic levels for the mechanism of *B. amyloliquefaciens* ZZ7 to synthesize PLO compounds, and provides a direction for the subsequent genetic modification of ZZ7 to solve PLO from the source in the MFB.

## 1. Introduction

Maotai flavor baijiu (MFB) is one of the four basic flavor baijiu in china, and is famous at home and abroad (Jin et al., 2017). MFB is brewed by open solid-state fermentation with grains as the main raw materials and high-temperature Daqu as the saccharification starter (Gan et al., 2019; Wang, 2022). The fermentation process involves many microorganisms, and the flavor compounds generated by their metabolism endow MFB with unique flavor and taste. Such as, phenylethanol produced by *Paecilomyces* makes it have elegant and aromatic flavor; the short chain fatty acid esters produced by *Monascus* spp make its taste soft and sweet (Wang et al., 2019; Xu et al., 2021). Up to now, more than 1000 volatile compounds have been identified in the flavor composition of MFB (Zhu et al., 2007; Liu and Sun, 2018). However, when the content of volatile compounds produced by microorganisms exceeds the standard or the proportion of volatile compounds is unbalanced, MFB will have defective styles. For example, the content of volatile sulfur compounds exceeds the standard or the ratio of volatile sulfur compounds is unbalanced, MFB will have defective styles of pickle like odor (PLO), which will seriously reduce the quality of MFB (Chen et al., 2017; Yang et al., 2019b).

In order to ensure the high-quality production of MFB, it is necessary to understand the formation mechanism of PLO compounds in the brewing process. In recent years, the flavor composition of PLO compounds has been deeply explored in the MFB. Yang et al. (2019a) found that the flavor compounds that causes the PLO are sulfur compounds. Furthermore, Wang L. et al. (2020) found that volatile sulfur compounds were significantly positively correlated with PLO in the MFB, such as dimethyl-disulfide, dimethyl-trisulfide and 2-furylthiol. In addition, the spatiotemporal distribution and accumulation of five volatile sulfur compounds that lead to the PLO were analyzed in the stacking fermentation process of MFB, and showed that some microorganisms in the piled fermented grains were correlated with the content of volatile sulfur compounds (Yang et al., 2023). These works have promoted our understanding of the composition of PLO components and their distribution and accumulation rules in the brewing process of MFB, which provided some guidance for scientifically regulating the production of MFB. However, these works did not identify the microorganisms that produce these volatile sulfur compounds.

In the brewing process of MFB, microorganisms will produce various flavor compounds or precursors, which will directly affect the sense and quality of MFB (Xu et al., 2023). Such as, pyrazine compounds synthesized by *Bacillus* contribute to their unique nutty flavor (Jin et al., 2019); the terpenes produced by *Mucor* make its taste more elegant and refined (Wang et al., 2015). The rapid development of systems biology has become an important means to explore the biological characteristics, gene function and metabolic regulation of unknown microorganisms (Fang et al., 2022; Zhang et al., 2023), and provide directions for the subsequent molecular genetic modification of microorganisms. For example, Wang et al. (2022) identified a *Lactobacillus pentosus* LTJ12 in MFB, and found that LTJ12 has multiple genes related to alcohol metabolism to enhance its ethanol tolerance and alcohol metabolism through whole genome sequencing and analysis; Liu et al. (2022) conducted genomic analysis on a caproic acid producing strain of *Rummeliibacillus suwonensis* 3B-1 isolated from Luzhou flavor baijiu, and excavated its biosynthesis pathway of caproic acid; Wu M. et al. (2023) used the whole gene sequencing technology to identify a new strain *Butyriproducens baijiuensis* BJN0003 that produced butyric acid isolated from the Chinese baijiu, and analyzed the butyric acid metabolic pathway of BJN0003 from glucose. The same is true for the PLO compounds, however, the metabolic pathway of PLO compounds in the microorganisms are still unclear in the MFB, and there are no relevant research reports. This makes it difficult to control the formation of PLO compounds in the brewing process of MFB from the source, and hinders the subsequent regulation of high-quality fermentation processes.

Based on the above analysis, in this study, we first isolated and identified *B. amyloliquefaciens* ZZ7, which metabolized typical flavor compounds of PLO from the fermented grains of MFB. Then, the synthesis ability of volatile sulfur compounds and physiological metabolic characteristics of *B. amyloliquefaciens* ZZ7 were analyzed based on metabolomics analysis, and the functional annotation of genome ZZ7 and an assessment of the metabolic pathways were performed by genome sequencing and annotation. Furthermore, we constructed the metabolic pathway of *B. amyloliquefaciens* ZZ7 to produce PLO compounds by combining metabolomics and genomics. This work not only revealed the main production strains of PLO compounds and its biological background, provided a reference for its genetic transformation, but also provided guidance for ensuring the high-quality production of MFB from the source.

## 2. Materials and methods

### 2.1. Samples, strain isolation, and cultivation conditions

The samples of fermented grains were collected from 1 to 7 rounds fermentation of Maotai flavor baijiu in Maotai Town, Zunyi City, Guizhou Province, numbered PA, PB, PC, PD, PE, PF, and PG groups, respectively. Then, take 1 g of the fermented grains sample and place it in 99 mL of sterile water. After diluting the sample appropriately, four samples of 100 microliters were spread on LB and PDA solid medium, respectively, and incubated at 32°C for 48 h. Next, single colonies were picked and cultured in PDA or LB liquid medium for 48 h at 32°C and 200 rpm/min. Finally, the sensory evaluation of the flavor characteristics of the single-strain fermentation broth was performed according to the previously reported method (He et al., 2021), to screen out the producing strains of PLO compounds.

### 2.2. Morphological observation and physiological and biochemical characteristics identification of strains

Morphology observation of strain: Inoculated strain ZZ7 onto LB solid culture medium and culture it at 37°C for 24 h; selected a single colony to dilute the smear, perform gram staining and observe the morphology of the colony. The cell morphology and staining conditions were observed and photographed under an optical microscope.

Physiological and biochemical characteristics identification of strain: According to the “Systematic Identification Manual of Common Bacteria” and the eighth edition of “Berger’s Manual of Bacterial Identification,” a total of six physiological and biochemical characteristics tests were conducted on strain ZZ7, including starch hydrolysis test, methyl red test, motility test, salt tolerance test, glycerol ketogenesis test and catalase test.

### 2.3. Phylogenetic analysis of strain ZZ7 with 16S rRNA

Genome of strain ZZ7 was extracted by DNA Extraction Kit (Solarbio, Beijing, China) according to standard protocol. The 16S rRNA of strain ZZ7 was amplified based on the primers 27F (5′-AGAGTTTGATCCTGGCTCAG-3′) and 1492R (5′-GGTACTTGTTACGACT-3′). Then, the 16S rRNA sequence obtained by sequencing was compared with the NCBI database, the phylogenetic analysis was carried out using MEGA X software (Kumar et al., 2018; Nouioui et al., 2018), and then the phylogenetic evolutionary tree of strain ZZ7 was constructed.

### 2.4. Genome sequence, prediction, and annotation

Whole-genome sequencing of *B. amyloliquefaciens* ZZ7 was analyzed through high throughput sequencing based on the Shanghai Majorbio Bio-Pharm Technology Co., Ltd. (Shanghai, China), and *de novo* assembly was performed based on SOAPdenovo (Li et al., 2010). Then, the general genetic information of *B. amyloliquefaciens* ZZ7 was predicted according to previously reported methods (Hao et al., 2023; Wu M. et al., 2023). Finally, Genes from *B. amyloliquefaciens* ZZ7 were annotated to the NR, SwissProt, COG, GO, and KEGG databases (Ashburner et al., 2000; Bairoch, 2000; Kanehisa, 2000; Jensen et al., 2007; Arthur et al., 2013). The genomic characteristics of *B. amyloliquefaciens* ZZ7 was analyzed by CGView software (Stothard and Wishart, 2005; Delcher et al., 2007). The numbers of carbohydrate-active enzymes (CAZy) of *B. amyloliquefaciens* ZZ7 were analyzed according to previously reported methods (Chen et al., 2021).

### 2.5. Metabolomic analysis of *Bacillus amyloliquefaciens* ZZ7

The volatile compounds in samples were analyzed by HS-SPME-GC-MS according to the previously reported methods (Wu J. et al., 2023). Samples (2 g) were mixed with NaCl saturated water solution, and ultrasonicated for 30 min. The internal standard (4-octanol, 5 mg/L of final concentration) was placed into a 15 mL vial and capped with a silicon septum tightly. The sample were extracted using a 50/30 μm DVB/CAR/PDMS fiber (Supelco, Bellefonte, PA, USA). The automatic headspace sampling system (Multi-Purpose Sample MPS 2 with a SPME adapter) (GERSTEL Inc., Baltimore, MD, United States) with a 50/30 mm DVB/CAR/PDMS fiber (Supelco Inc., Bellefonte, PA, United States) was used in SPME. Samples were preheated for 5 min and extracted for 45 min at 40°C. The GC-MS condition was a starting temperature of 40°C, then increased to 230°C at a rate of 5°C/min and held at 230°C for 20 min. The flow rate of a helium carrier gas was 2 mL/min. Mass spectrometry (MS) was generated with electron impact of 70 eV ionization energy and a full scan range from 35 to 500 amu. Their spectral data of volatile compounds were compared with published values and further confirmed using NIST/EPA/NIH Mass spectral database (NIST11) with NIST MS search program v.2.0 g (Zhu et al., 2018). Finally, we used MassHunter software to process the raw data after GC-MS analysis for qualitative and quantitative analysis of volatile compounds.

The non-volatile compounds in samples were analyzed by LC-MS. A 150 μL extract solution (ACN: Methanol = 1:4, V/V) containing internal standard (2-chloro-L-phenylalanine) was added into 50 μL sample, the sample was vortex for 3 min and centrifuged at 12,000 rpm for 10 min, a 150 μL aliquots of the supernatant was colleted and placed in −20°C for 30 min, and then a 120 μL aliquots of supernatant were transferred for LC-MS analysis.

HPLC conditions: Waters ACQUITY UPLC BEH C18 (1.8 μm, 2.1 mm × 100 mm); column temperature is 40°C; flow rate is 0.4 mL/min; injection volum is 2 μL; solvent system is water (0.1% formic acid): acetonitrile (0.1% formic acid); The column was eluted with 5% mobile phase B (0.1% formic acid in acetonitrile) at 0 min followed by a linear gradient to 90% mobile phase B (0.1% formic acid in acetonitrile) over 11 min, held for 1 min, and then come back to 5% mobile phase B within 0.1 min, held for 1.9 min, then rapidly return to starting conditions.

Mass spectrometry conditions: Electrospray ionization (ESI) source, negative ion mode scanning; desolvation line (DL) temperature was 250°C; heating module temperature was 200°C; nitrogen atomization gas flow rate was 1.5 L/min. Qualitatively adopts the full scan mode (Full Scan), and the mass scan range is 100∼300 m/z. Based on the quantitative ions screened by the Scan mode, the selected ion monitoring (SIM) scanning mode is used to quantitatively analyze non-volatile compounds.

### 2.6. Statistical analysis

The SPSS software was used for statistical analyzing the contents of volatile compounds from GC-MS detection and other data, including alcohols, acids, esters, phenols, pyrazines, sulfur compounds, nitrogen compounds, terpenoids and other compounds, etc. All analyses were repeated in triplicate.

## 3. Results and discussion

### 3.1. Isolation and physiological characteristics of PLO compounds production strains

**FIGURE 1:**
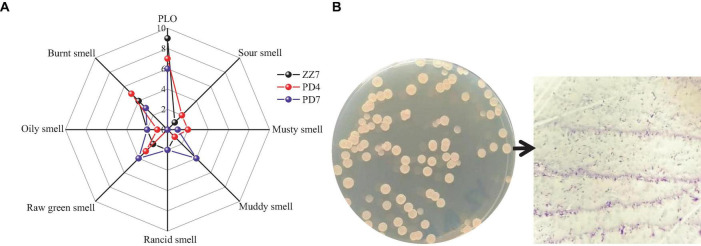
Radar image of sensory evaluation of ZZ7, PD4, and PD7 strains **(A)**; morphological characteristics and gram staining of ZZ7 strains **(B)**.

In order to reveal the main microorganisms that produce PLO compounds, we obtained 103 strains from the fermented grains of Maotai flavor baijiu (MFB) with pickle like odor (PLO) through microbial isolation, purification and culture. Then, shake flask fermentation was carried out on these strains, and the sensory evaluation of PLO was carried out on 103 groups of single-strain fermentation broths in turn. During this process, we screened out three strains ZZ7, PD4, and PD7 with typical PLO characteristics. Then, based on the odor flavor wheel of MFB (PLO, sour smell, musty smell, muddy smell, rancid smell, oily smell, raw green smell, burnt smell), we used a 10-point method (0 means no flavor perception, 10 represents the strongest flavor perception) to evaluate the odor flavor of these strains to obtain a radar map of strain scores with typical PLO characteristics (Figure 1A). The results showed that the PLO characteristics of strain ZZ7 were the most obvious, so it was presumed that it just a class of strains that primarily produce PLO compounds in MFB.

Furthermore, we found that the colony morphology of strain ZZ7 is circular and smooth, milky white, with a slight protrusion in the middle, neat edges, opaque, uniform size and is a gram-positive bacterium through morphological observation and gram staining (Figure 1B). In addition, through the identification of six physiological and biochemical characteristics of starch hydrolysis, methyl red, motility, salt tolerance, glycerol ketogenesis and catalase, it was showed that strain ZZ7 has starch hydrolysis ability, catalase activity and motility, as well as the ability to convert glycerol to acetone. It has low acid production from glucose decomposition during glucose metabolism, and can grow normally under the condition of 2–5% NaCl, but cannot grow normally under the condition of 7% NaCl (Table 1).

**TABLE 1:** Different physiological characteristics of strain ZZ7 results.

Physiological characteristics	Results
Starch hydrolysis	+
Methyl red	−
Motility	+
Salt tolerance	+
Glycerol ketogenesis	+
Catalase	+

+ Indicates positive reaction.

− Indicates negative reaction.

### 3.2. Species identification and phylogenetic analysis of PLO producing strain ZZ7

**FIGURE 2:**
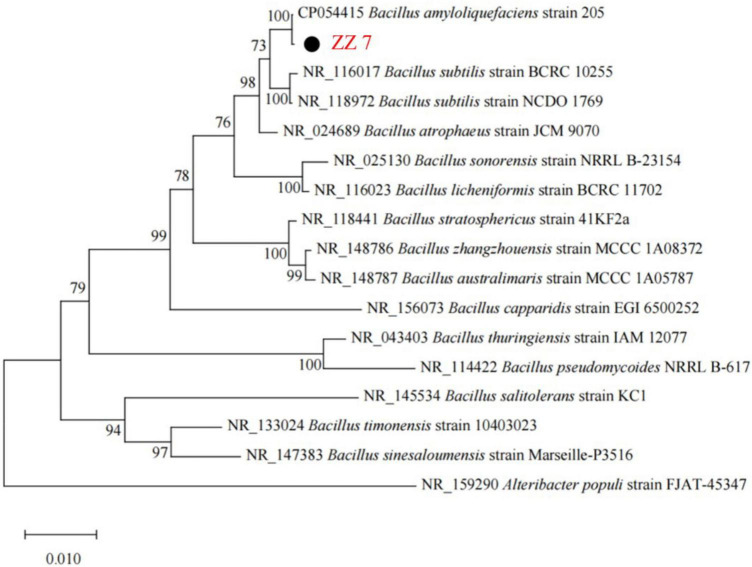
Phylogenetic tree of strain ZZ7.

Based on the above results, the species identification of strain ZZ7 was conducted. Firstly, we extracted the genomic DNA of strain ZZ7. Then, a phylogenetic tree of strain ZZ7 was constructed after polymerase chain reaction (PCR) amplification, sequencing and blast analysis of 16S rRNA (Figure 2 and Supplementary Table 1). The results showed that strain ZZ7 had the closest relationship with *B. amyloliquefaciens*, and its sequence similarity with *B. amyloliquefaciens* 205 on the same branch was 100%, so we could determine that strain ZZ7 was *B. amyloliquefaciens*.

It was previously reported that co-cultivation of *Bacillus licheniformis* and *Zygosaccharomyces* promoted the synthesis of sulfur compounds during the brewing of MFB (Xu et al., 2018). Here, we are the first to identify a strain of *B. amyloliquefaciens* ZZ7 that produces typical PLO compounds. As we all know, *Bacillus* is the dominant bacteria in the brewing process of MFB, and its secreted amylase, protease, pyrazine and other flavor compounds are extremely important to the production of high-quality MFB (Zhu et al., 2009). This implied that the content of volatile sulfur compounds in *B. amyloliquefaciens* ZZ7 needs to be reduced through molecular genetic transformation, thus ensuring the production quality of MFB from the source.

### 3.3. Metabolic phenotype analysis of *Bacillus amyloliquefaciens* ZZ7

**FIGURE 3:**
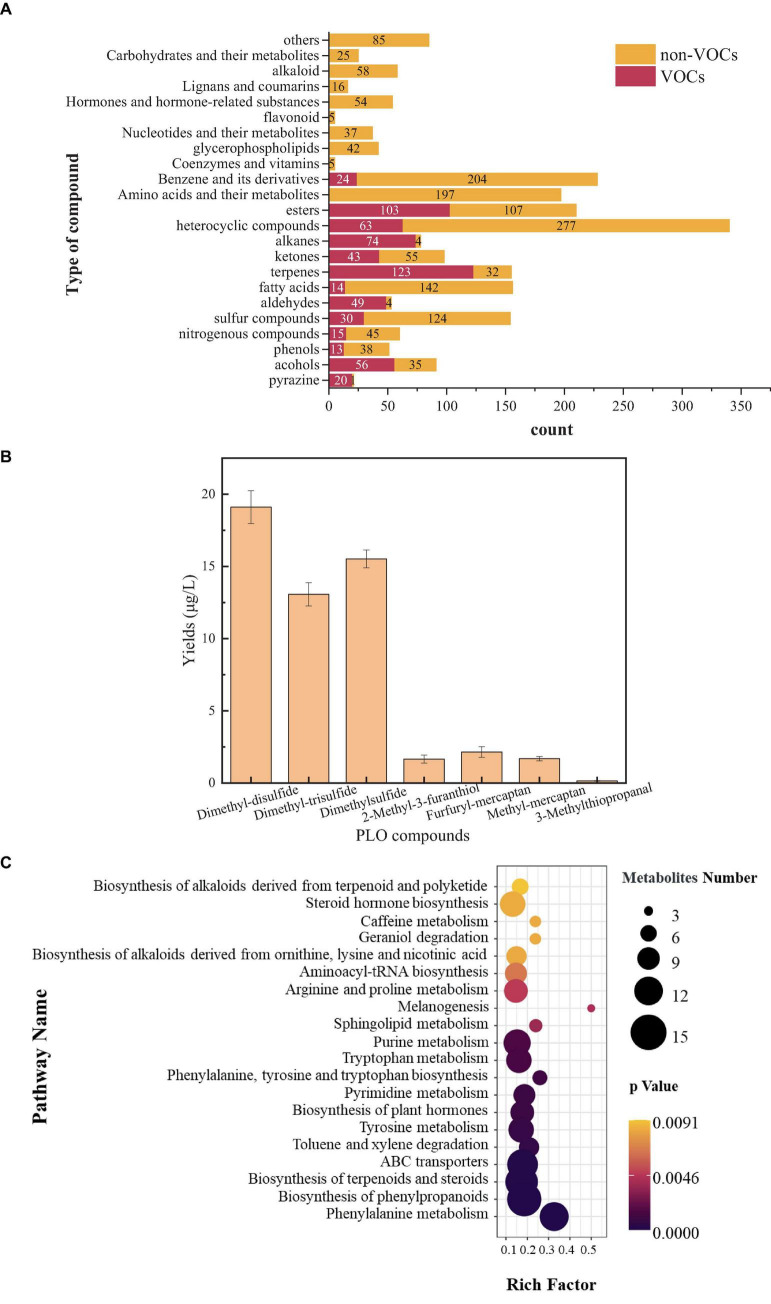
Detection of volatile and non-volatile compounds in *Bacillus amyloliquefaciens* ZZ7 **(A)**; types and content of sulfur compounds in *Bacillus amyloliquefaciens* ZZ7 **(B)**; enrichment map of metabolic pathways in *Bacillus amyloliquefaciens* ZZ7 **(C)**.

To analyze the type and content of PLO compounds produced by *B. amyloliquefaciens* ZZ7, as well as its metabolic phenotype. Firstly, The HS-SPME-GC-MS was used to detect and analyze volatile compounds in the fermentation broth of *B. amyloliquefaciens* ZZ7, we detected 13 types of volatile compounds, including alcohols, acids, esters, phenols, pyrazines, sulfur compounds, nitrogen compounds, terpenoids, and other compounds, with a total of 627 kinds of volatile compounds. It contains 56 kinds of alcohols, 14 kinds of acids, 103 kinds of esters, 13 kinds of phenols, 20 kinds of pyrazines, 30 kinds of sulfur compounds, 15 kinds of nitrogen compounds, 123 kinds of terpenoids, 49 kinds of aldehydes, 43 kinds of ketones, 74 kinds of alkanes, 63 kinds of heterocyclic compounds, 24 kinds of benzene and its derivatives (Figure 3A).

Among them, some volatile compounds have a significant contribution to the flavor of MFB, such as 2,3-butanediol with cream and butter aroma, its content is 274.99 μg/L; and ethyl phenylacetate with rose and honey aroma, its content reaches 6.15 μg/L, trimethyl-pyrazine with a nutty taste, its content reaches 0.78 μg/L, etc (Supplementary Table 2). These results showed that *B. amyloliquefaciens* ZZ7 not only produced typical PLO compounds, but also synthesized many flavor compounds that contributed to the unique flavor of MFB. The non-volatile compounds were also detected and analyzed based on the LC-MS. It contains more types and quantities of compounds compared to volatile compounds, totaling 23 types and 1,507 kinds of non-volatile compounds (Figure 3A).

Next, we identified and quantitatively analyzed the volatile sulfur compounds that can lead to the PLO, and found that the total content of volatile sulfur compounds produced by *B. amyloliquefaciens* ZZ7 was 53.34 μg/L (Figure 3B). Among them, there are 7 volatile sulfur compounds that can cause PLO, including dimethyl-disulfide (odor: salty and onion), dimethyl-trisulfide (odor: salty), dimethyl-sulfur (odor: sulfur and onion), *B. amyloliquefaciens* ZZ7 was 53.34 μg/L (Figure 3B). Among them, there are 7 volatile sulfur compounds that can cause PLO, including dimethyl-disulfide (odor: salty and onion), dimethyl-trisulfide (odor: salty), dimethyl-sulfur (odor: sulfur and onion), 2-methyl-3-furanthiol (odor: sulfur and fishy), furfuryl mercaptan (odor: sulfur and bacon), methyl mercaptan (odor: sulfur and onion), and 3-methylthiopropanal (odor: onion and broth). Their contents were 19.11, 13.07, 15.52, 1.66, 2.15, 1.69, and 0.15 μg/L (Figure 3B). The results showed that the contents of dimethyl-disulfide, dimethyl-trisulfide and dimethyl-sulfur accounted for a relatively high proportion of the total content of volatile sulfur compounds, respectively 35.83, 24.50, and 29.10%, which indicated that the content of three compounds of *B. amyloliquefaciens* ZZ7 should be mainly controlled in the brewing process of MFB. In addition, these sulfur compounds alone failed to present PLO, such as the flavor characteristics of dimethyl disulfide are salty and onion; the flavor characteristics of dimethylsulfide are sulfur and onion; the flavor characteristics of dimethyl trisulfide are salty taste (Sha et al., 2016), but the overall presents typical PLO characteristics, which is consistent with the joint action of various sulfur compounds produce the PLO of MFB.

Based on the types of volatile and non-volatile compounds of *B. amyloliquefaciens* ZZ7 and KEGG database, the metabolic characteristics of *B. amyloliquefaciens* ZZ7 was analyzed. It mainly participates in the metabolic pathways of phenylalanine metabolism, biosynthesis of phenylpropanoids, terpenoids, and steroids, while hardly participating in the melanogenesis metabolic pathway (Figure 3C). To sum up, these results showed that *B. amyloliquefaciens* ZZ7 that produced PLO compounds, has a strong ability to metabolize aromatic compounds and terpenoids and steroids, these compounds are essential for MFB. It again showed that the content of volatile sulfur compounds can only be reduced through genetic transformation of *B. amyloliquefaciens* ZZ7, thus ensuring the high-quality production of MFB from the source.

### 3.4. Whole genome sequencing and analysis of *Bacillus amyloliquefaciens* ZZ7

**FIGURE 4:**
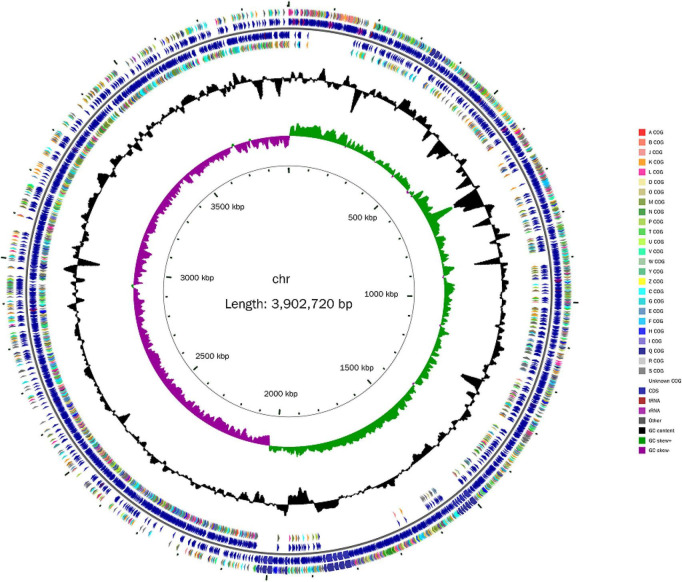
Genome map of *Bacillus amyloliquefaciens* ZZ7.

Next, the whole genome of *B. amyloliquefaciens* ZZ7 was sequenced and annotated. It showed that the genome size of *B. amyloliquefaciens* ZZ7 was 3,902,720 bp, with a GC content of 46.09%, and a total of 3,948 protein coding genes were predicted. Moreover, it also includes 27 rRNAs (9 5S rRNAs, 9 16S rRNAs, 9 23S rRNAs), 86 tRNAs, 81 ncRNAs, and 0 CRISPRs structures (Figure 4 and Table 2). Furthermore, the predicted coding genes of *B. amyloliquefaciens* ZZ7 were compared and analyzed with NR, eggNOG, GO, KEGG and SwissProt databases, respectively. The annotated number of genes was the highest in the NR database, with 3,913, accounting for 99.11% of the total predicted genes, and the sequences of genes annotated had the highest similarity with the genome sequence of *B. amyloliquefaciens*, reaching 100%, this indicated that the strain ZZ7 is highly similar to *B. amyloliquefaciens* in terms of genome structure and homology, it is also consistent with the results of 16S rRNA gene classification and identification mentioned earlier. The remaining databases were SwissProt, eggNOG, GO, and KEGG, with the annotated number of genes accounting for 89.28, 84.80, 68.92, and 54.02% of the total predicted genes (3948 protein coding genes), respectively (Table 2).

**TABLE 2:** Genome characteristics of *Bacillus amyloliquefaciens* ZZ7.

Characteristics	Number
Genome size (bp)	3902720
GC content (%)	46.09%
Gene (CDS) no.	3948
rRNA no.	27
5S rRNA	9
16S rRNA	9
23S rRNA	9
tRNA no.	86
nc RNA no.	81
CRISPRs no.	0
NR annotation	3913
eggNOG annotation	3348
GO annotation	2721
SwissProt annotation	3525
KEGG annotation	2133

### 3.5. Functional annotation and analysis of genome of *Bacillus amyloliquefaciens* ZZ7

**FIGURE 5:**
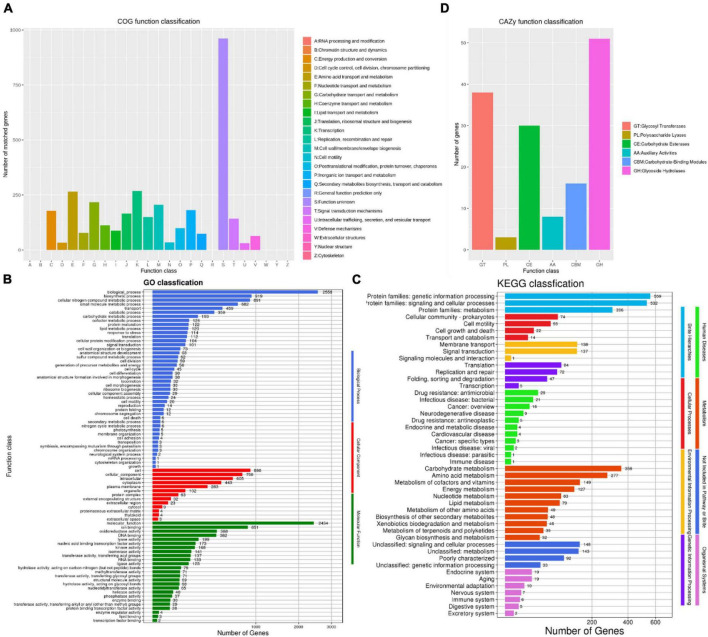
Functional annotation and analysis of genome of *Bacillus amyloliquefaciens* ZZ7. The COG database annotation of *B. amyloliquefaciens* ZZ7 genome **(A)**; the GO database annotation of *B. amyloliquefaciens* ZZ7 genome **(B)**; the KEGG database annotation of *Bs* ZZ7 genome **(C)**; the carbohydrate active enzymes annotation of *B. amyloliquefaciens* ZZ7 genome **(D)**.

The number of COG types was 19, with a total of 3,348 protein coding genes was annotated in the COG database, accounting for 84.80% of the annotated genes (Figure 5A). Among them, a total of 268 genes (6.78%) were associated with transcription, 265 genes (6.71%) were associated with amino acid transport and metabolism, 217 genes (5.49%) were related to carbohydrate transport and metabolism, 206 genes (5.21%) were associated with the generation of cell wall/membrane, 181 genes (4.58%) were associated with inorganic ion transport and metabolism, and 961 unknown functional factors, accounting for 24.34%. This also indicated that there are many unknown gene functions need further exploration in *B. amyloliquefaciens* ZZ7.

There are three main categories in the GO database, includes molecular function, cellular components and biological processes (Ashburner et al., 2000). In the GO database, a total of 2,721 genes were annotated, accounting for 68.92% of the total annotated genes (Figure 5B). Under the three major categories, it can be further divided into 82 types of functional annotations. The biological process contains 44 branches, among which the four pathways of biological process, biosynthesis process, cell nitrogen compound metabolic process and small molecule metabolic process have the most enriched genes, with 2556, 919, 891, and 682 genes, respectively; the cellular components includes 13 branches, among which the number of genes enriched in cells (898 genes), cellular components (758 genes), and intracellular (605 genes) is the largest; while the molecular function includes 25 branches, the number of related genes involved in the ion-binding pathway is the largest, with 851 (Figure 5B).

In the KEGG database, a total of 2,133 genes were annotated in eight categories, including human diseases, metabolism, genetic information processing, cellular processes, etc., accounting for 54.02% of the total annotated genes (Figure 5C). The results show that the number of genes enriched in metabolism is the largest, with a total of 1,287 genes, among which genes related to carbohydrate metabolism, amino acid metabolism, cofactor and vitamin metabolism and energy metabolism pathways account for a relatively high proportion, with 358, 277, 149, and 127 genes, respectively. It showed that *B. amyloliquefaciens* ZZ7 has carbohydrate metabolism ability to produce metabolites and secondary metabolites.

Finally, the carbohydrate active enzymes (CAZy) annotation of *B. amyloliquefaciens* ZZ7 was performed. It showed that a total of 146 genes were identified, including 51 glycoside hydrolases, 38 glycosyltransferases, 3 polysaccharide lyases, 30 carbohydrate esterases, 8 auxiliary active enzymes and 16 enzymes related to carbohydrate binding modules (Figure 5D). In summary, these results revealed the genetic background and biological characteristics of *B. amyloliquefaciens* ZZ7, which provide a reference and guidance for its genetic modification.

### 3.6. Analysis of sulfur compounds metabolic pathways of *Bacillus amyloliquefaciens* ZZ7

In the Maotai flavor baijiu, the type and content of volatile sulfur compounds have a significant impact on its flavor characteristics. There are two ways to form volatile sulfur compounds, including microbial metabolism and chemical reactions. Microbial metabolism mainly involves the degradation of proteins in raw materials into sulfur-containing amino acids (cystine and cysteine) by microorganisms under the action of various enzymes, the sulfur-containing amino acids continue to degrade to produce sulfur compounds, such as hydrogen sulfide, 3-methylthiopropanal, etc. The chemical reaction is mainly the maillard reaction between some sulfur-containing amino acids and carbohydrates to produce furyl mercaptan, 2-methyl-3-furan mercaptan, etc (Yang et al., 2019a; Wang N. et al., 2020).

**FIGURE 6:**
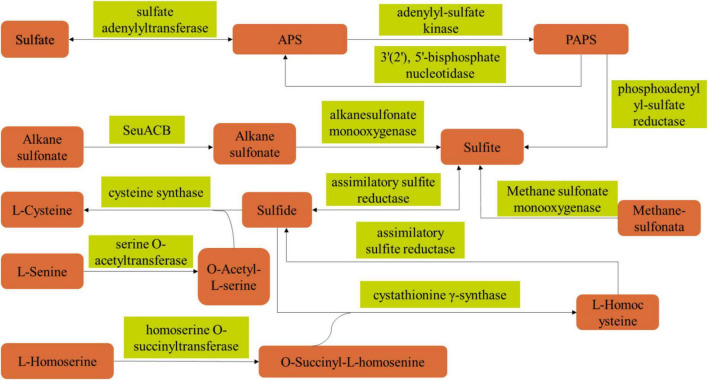
Metabolic pathway of sulfur compounds in *B. amyloliquefaciens* ZZ7.

Based on the above results, in order to elucidate the metabolic synthesis pathway of PLO compounds of *B. amyloliquefaciens* ZZ7, we analyzed the volatile sulfur compounds metabolic pathway of *B. amyloliquefaciens* ZZ7 by combining genomics and metabolomics analysis data (Figure 6 and Supplementary Table 3). The results showed that 12 enzymes were involved the metabolic synthetic pathways of volatile sulfur compounds in *B. amyloliquefaciens* ZZ7, including sulfate adenylyltransferase, cysteine-synthase, homoserine O-succinyltransferase, assimilative-sulfite reductase and alkanesulfonic acid monooxygenase, etc (Figure 6). Among them, 4 enzymes were involved in the sulfate reduction pathway, including adenosyl sulfate transferase (EC 2.7.7.4), adenosyl sulfate kinase (EC 2.7.1.25), adenosyl phosphate sulfate reductase (EC 1.8.4.8) and assimilating sulfite reductase (EC 1.8.1.2); 3 enzymes, serine O-acetyltransferase (EC 2.3.1.30), cysteine synthase (EC 2.5.1.47), cystathionine γ-synthase (EC 2.5.1.48) are involved in the cysteine metabolic pathway; while homoserine O-succinyltransferase (EC 2.3.1.46) involves in the methionine metabolism pathway (Supplementary Table 2). It not only proved that *B. amyloliquefaciens* ZZ7 had the ability to produce PLO compounds, but also provided a reference for genetic transformation of *B. amyloliquefaciens* ZZ7 to regulate the content of volatile sulfur compounds in the MFB brewing system. Moreover, for these 12 enzymes, we can choose different weak expression elements (such as weak promoter, weak RBS, weak terminator) to replace the natural expression elements of the 12 enzymes, respectively, thereby reducing the synthesis levels of these enzymes, to achieve the goal of reducing the accumulation of PLO compounds in *B. amyloliquefaciens* ZZ7.

## 4. Conclusion

Revealing the production strain of PLO compounds is to ensure the high-quality production of MFB from the source. In this study, we first screened and identified a *B. amyloliquefaciens* ZZ7 with high content of PLO compounds in fermented grains of MFB. There are 7 volatile sulfur compounds produced by *B. amyloliquefaciens* ZZ7 that cause PLO, including dimethyl-sulfur, dimethyl-disulfide, dimethyl-trisulfide, 2-methyl-3-furan mercaptan, 3-methylthiopropanal, methyl mercaptan and furfuran mercaptan. The genome size of *B. amyloliquefaciens* ZZ7 is 3,902,720 bp, with a GC content of 46.09%, and a total of 3,948 protein coding genes were predicted. Moreover, there are a total of 2,134 volatile compounds and non-volatile compounds metabolized by *B. amyloliquefaciens* ZZ7, which mainly involved in the transport and metabolism of amino acids, carbohydrates and biological processes. the PLO compounds metabolic pathway of *B. amyloliquefaciens* ZZ7 was constructed by combining genomics and metabolomics analysis data. This study reveals the main production strain (*B. amyloliquefaciens* ZZ7) of PLO compounds, its biological background and PLO compounds metabolic pathway, which provides a reference for its genetic transformation, enriches the genomics research of MFB-brewing microorganisms. Simultaneously, it provides guidance for controlling the formation of pickle like odor of Maotai flavor baijiu from the source.

## Data availability statement

The original contributions presented in the study are included in the article/Supplementary material, further inquiries can be directed to the corresponding author.

## Author contributions

LY: Funding acquisition, Writing – original draft, Writing – review and editing. SZ: Data curation, Writing – original draft. MZ: Data curation, Formal analysis, Writing – original draft. YL: Data curation, Methodology, Writing – original draft. ZJ: Funding acquisition, Resources, Writing – review and editing. PC: Conceptualization, Resources, Writing – original draft. CZ: Funding acquisition, Resources, Writing – review and editing.

## References

[B1] Arthur J. W. Yu K. Zhang T. (2013). Construction of customized sub-databases from NCBI-nr database for rapid annotation of huge metagenomic datasets using a combined BLAST and MEGAN approach. *PLoS One* 8:e59831. 10.1371/journal.pone.0059831 23573212 PMC3613424

[B2] Ashburner M. Ball C. A. Blake J. A. Botstein D. Butler H. Cherry J. M. (2000). Gene ontology: Tool for the unification of biology. *Nat. Genet.* 25 25–29. 10.1038/75556 10802651 PMC3037419

[B3] Bairoch A. R. (2000). The SWISS-PROT protein sequence database and its supplement TrEMBL in 2000. *Nucleic Acids Res.* 28 45–48. 10.1093/nar/28.1.45 10592178 PMC102476

[B4] Chen S. Sha S. Qian M. Xu Y. (2017). Characterization of volatile sulfur compounds in Moutai liquors by headspace solid-phase microextraction gas chromatography-pulsed flame photometric detection and odor activity value. *J. Food Sci.* 82 2816–2822. 10.1111/1750-3841.13969 29131338

[B5] Chen Y. Zhao S. Zhang C. Qiao N. Duan H. Xiao Y. (2021). Integrated phenotypic–genotypic analysis of *Latilactobacillus sakei* from different niches. *Foods* 10:1717. 10.3390/foods10081717 34441495 PMC8393274

[B6] Delcher A. L. Bratke K. A. Powers E. C. Salzberg S. L. (2007). Identifying bacterial genes and endosymbiont DNA with Glimmer. *Bioinformatics* 23 673–679. 10.1093/bioinformatics/btm009 17237039 PMC2387122

[B7] Fang G. Y. Chai L. J. Zhong X. Z. Lu Z. M. Zhang X. J. Wu LH. (2022). Comparative genomics unveils the habitat adaptation and metabolic profiles of clostridium in an artificial ecosystem for liquor production. *mSystems* 7:e0029722. 10.1128/msystems.00297-22 35491831 PMC9238394

[B8] Gan S. H. Yang F. Sa S. K. Luo R. Y. Liao S. L. Wang H. Y. (2019). Deciphering the composition and functional profile of the microbial communities in Chinese Moutai liquor starters. *Front. Microbiol.* 10:1540. 10.3389/fmicb.2019.01540 31333631 PMC6620787

[B9] Hao S. Ren Q. Wang J. Li L. Huang M. (2023). Two novel planococcus species isolated from baijiu pit mud with potential application in brewing. *Front. Microbiol.* 14:1139810. 10.3389/fmicb.2023.1139810 37250023 PMC10213732

[B10] He Y. Tang K. Yu X. Chen S. Xu Y. (2021). Identification of compounds contributing to trigeminal pungency of baijiu by sensory evaluation, quantitative measurements, correlation analysis, and sensory verification testing. *J. Agr. Food Chem* 70 598–606. 10.1021/acs.jafc.1c06875 34939413

[B11] Jensen L. J. Julien P. Kuhn M. Muller J. Doerks T. Bork P. (2007). eggNOG: Automated construction and annotation of orthologous groups of genes. *Nucleic Acids Res.* 36 250–254. 10.1093/nar/gkm796 17942413 PMC2238944

[B12] Jin G. Zhu Y. Xu Y. (2017). Mystery behind Chinese liquor fermentation. *Trends Food Sci. Tech.* 63 18–28. 10.3389/fmicb.2019.00696 31031717 PMC6473189

[B13] Jin Y. Li D. Ai M. Tang Q. Huang J. Ding X. (2019). Correlation between volatile profiles and microbial communities: A metabonomic approach to study Jiang-flavor liquor Daqu. *Food Res. Int.* 121 422–432. 10.1016/j.foodres.2019.03.021 31108766

[B14] Kanehisa G. S. (2000). KEGG: Kyoto encyclopedia of genes and genomes. *Nucleic Acids Res.* 28 27–30. 10.1093/nar/28.1.27 10592173 PMC102409

[B15] Kumar S. Stecher G. Li M. Knyaz C. Tamura K. Battistuzzi F. U. (2018). MEGA X: Molecular evolutionary genetics analysis across computing platforms. *Mol. Biol. Evol.* 35 1547–1549. 10.1093/molbev/msy096 29722887 PMC5967553

[B16] Li R. Zhu H. Ruan J. Qian W. Fang X. Shi Z. (2010). De novo assembly of human genomes with massively parallel short read sequencing. *Genome Res.* 20 265–272. 10.1101/gr.097261.109 20019144 PMC2813482

[B17] Liu C. Du Y. Zheng J. Qiao Z. Luo H. Zou W. (2022). Production of caproic acid by *Rummeliibacillus suwonensis* 3B-1 isolated from the pit mud of Strong-flavor baijiu. *J. Biotechnol.* 358 33–40. 10.1016/j.jbiotec.2022.08.017 36049550

[B18] Liu H. Sun B. (2018). Effect of fermentation processing on the flavor of baijiu. *J. Agr. Food Chem.* 66 5425–5432. 10.1021/acs.jafc.8b00692 29751730

[B19] Nouioui I. Carro L. García L. M. Meier J. P. Woyke T. Kyrpides N. C. (2018). Genome-based taxonomic classification of the phylum actinobacteria. *Front. Microbiol.* 9:2007. 10.3389/fmicb.2018.02007 30186281 PMC6113628

[B20] Sha S. Chen S. Qian M. Wang C. Xu Y. (2016). Characterization of the typical potent odorants in Chinese roasted sesame-like flavor type liquor by headspace solid phase microextraction–aroma extract dilution analysis, with special emphasis on sulfur-containing odorants. *J. Agr. Food Chem* 65 123–131. 10.1021/acs.jafc.6b04242 27989125

[B21] Stothard P. Wishart D. S. (2005). Circular genome visualization and exploration using CGView. *Bioinformatics* 21 537–539. 10.1093/bioinformatics/bti054 15479716

[B22] Wang H. Y. Zhang Q. L. Du G. C. Yang F. Li J. H. (2019). Whole-genome sequencing and analysis of *Paecilomyces variotii* MTDF-01, isolated during Maotai-flavor liquor brewing. *Food Sci.* 40 185–192.

[B23] Wang J. Lu C. Xu Q. Li Z. Song Y. Zhou S. (2022). Comparative genomics analysis provides new insights into high ethanol tolerance of *Lactiplantibacillus pentosus* LTJ12, a novel strain isolated from Chinese baijiu. *Foods* 12:35. 10.3390/foods12010035 36613254 PMC9818588

[B24] Wang L. (2022). Research trends in Jiang-flavor baijiu fermentation: From fermentation microecology to environmental ecology. *J. Food Sci.* 87 1362–1374. 10.1111/1750-3841.16092 35275413

[B25] Wang L. Fan S. Yan Y. Yang L. Chen S. Xu Y. (2020). Characterization of potent odorants causing a pickle-like off-odor in Moutai-aroma type baijiu by comparative aroma extract dilution analysis, quantitative measurements, aroma addition, and omission studies. *J. Agr. Food Chem* 68 1666–1677. 10.1021/acs.jafc.9b07238 31957444

[B26] Wang L. Hu G. Lei L. Lin L. Wang D. Wu J. (2015). Identification and aroma impact of volatile terpenes in Moutai liquor. *Int. J. Food Prop.* 19 1335–1352. 10.1080/10942912.2015.1064442

[B27] Wang N. Saidhareddy P. Jiang X. (2020). Construction of sulfur-containing moieties in the total synthesis of natural products. *Nat. Prod. Rep.* 37 246–275. 10.1039/c8np00093j 31204423

[B28] Wu J. Chen R. Li X. Fu Z. Xian C. Zhao W. (2023). Comprehensive identification of key compounds in different quality grades of soy sauce-aroma type baijiu by HS-SPME-GC-MS coupled with electronic nose. *Front. Nutr.* 10:1132527. 10.3389/fnut.2023.1132527 36960200 PMC10028209

[B29] Wu M. Xu Y. Dai M. Li W. Zhang C. Li X. (2023). *Butyriproducens baijiuensis* BJN0003: A potential new member of the family *Oscillospiraceae* isolated from Chinese baijiu. *3 Biotech* 13:205. 10.1007/s13205-023-03624-w 37223001 PMC10200727

[B30] Xu Y. Wang Q. Xu Y. (2018). Effects of main functional strains on *Zygosaccharomyces bailii* in Chinese Maotai-flavor liquor fermentation. *Microbiol. China* 45 42–53.

[B31] Xu Y. Wang X. Liu X. Li X. Zhang C. Li W. (2021). Discovery and development of a novel short-chain fatty acid ester synthetic biocatalyst under aqueous phase from *Monascus purpureus* isolated from Baijiu. *Food Chem* 338:128025. 10.1016/j.foodchem.2020.128025 32927200

[B32] Xu Y. Wu M. Zhao D. Zheng J. Dai M. Li X. (2023). Simulated fermentation of Strong-flavor baijiu through functional microbial combination to realize the stable synthesis of important flavor chemicals. *Foods* 12:644. 10.3390/foods12030644 36766173 PMC9913964

[B33] Yang L. Chen R. Liu C. Chen L. Yang F. Wang L. (2023). Spatiotemporal accumulation differences of volatile compounds and bacteria metabolizing pickle like odor compounds during stacking fermentation of Maotai-flavor baijiu. *Food Chem* 426:136668. 10.1016/j.foodchem.2023.136668 37356241

[B34] Yang L. Chen S. Xu Y. (2019a). Identification of volatile compounds from pickled mustard like off flavor Maotai by LLE and HS-SPME combined with GC-MS. *Food Ferm. Ind.* 45 221–226.

[B35] Yang L. Luo C. X. Zhang C. L. (2019b). Analysis of volatile flavor compounds from defective product of Sauce-flavor baijiu by GC×GC-TOF-MS. *China Brewing* 38 67–72.

[B36] Zhang G. Xiao P. Xu Y. Li H. Li H. Sun J. (2023). Isolation and characterization of *yeast* with benzenemethanethiol synthesis ability isolated from baijiu Daqu. *Foods* 12:2464. 10.3390/foods12132464 37444202 PMC10340341

[B37] Zhu B. F. Xu Y. Fan W. L. (2009). High-yield fermentative preparation of tetramethylpyrazine by *Bacillus* sp. using an endogenous precursor approach. *J. Industr. Microbiol. Biotechnol.* 37 179–186. 10.1007/s10295-009-0661-5 19904566

[B38] Zhu S. Lu X. Ji K. Guo K. Li Y. Wu C. (2007). Characterization of flavor compounds in Chinese liquor Moutai by comprehensive two dimensional gas chromatography/time of flight mass spectrometry. *Anal. Chim. Acta* 597 340–348. 10.1016/j.aca.2007.07.007 17683748

[B39] Zhu Y. Z. Liu J. W. Wang X. Jeong I. H. Ahn Y. J. Zhang C. J. (2018). Anti-BACE1 and antimicrobial activities of steroidal compounds isolated from marine Urechis unicinctus. *Mar. Drugs* 16 94 10.3390/md16030094 29538306 PMC5867638

