# Supplementary material for: Comprehensive genomic and metabolomic analysis revealed the physiological characteristics and pickle like odor compounds metabolic pathways of Bacillus amyloliquefaciens ZZ7 isolated from fermented grains of Maotai-flavor baijiu
Source: Front Microbiol. 2023 Oct 30;14:1295393. doi: 10.3389/fmicb.2023.1295393 (PMC10642760; doi:10.3389/fmicb.2023.1295393)
Supplement: Supplementary file 1 [file Data_Sheet_1.docx]

**Supplementary materials for**

**Comprehensive genomic and metabolomic analysis revealed the physiological characteristics and pickle like odor compounds metabolic pathways of *Bacillus amyloliquefaciens* ZZ7 isolated from Maotai-flavor baijiu**

Liang Yang^1^, Shuangran Zeng^1^, Meidi Zhou^1^, Yuetao Li^1,2^, Zeyuan Jiang^1^, Pingyan Cheng^3^, Chunlin Zhang^1^*

*^1^* *Department of Brewing engineering,* *Moutai Institute, Renhuai 564501, China.*

*^2^ College of Life Sciences, Shihezi University, Shihezi 832003, China*

*^3^ Guizhou Xijiu Co., Ltd.*

^*^Corresponding author: Prof. C. Zhang

Tel./fax.: +86-0851-28797035

E-mail address: zcl818075@163.com (C. Zhang)

Postal address: Department of Brewing engineering, Moutai Institute, Renhuai 564501, China

Table **S1.** Sequence of 16S rRNA of *Bacillus amyloliquefaciens* ZZ7.

| rRNA | Sequences |
| --- | --- |
| 16S | CATACATGCAAGTCGAGCGGACAGATGGGAGCTTGCTCCCTGATGTTAGCGGCGGACGGGTGAGTAACACGTGGGTAACCTGCCTGTAAGACTGGGATAACTCCGGGAAACCGGGGCTAATACCGGATGCTTGTTTGAACCGCATGGTTCAGACATAAAAGGTGGCTTCGGCTACCACTTACAGATGGACCCGCGGCGCATTAGCTAGTTGGTGAGGTAACGGCTCACCAAGGCGACGATGCGTAGCCGACCTGAGAGGGTGATCGGCCACACTGGGACTGAGACACGGCCCAGACTCCTACGGGAGGCAGCAGTAGGGAATCTTCCGCAATGGACGAAAGTCTGACGGAGCAACGCCGCGTGAGTGATGAAGGTTTTCGGATCGTAAAGCTCTGTTGTTAGGGAAGAACAAGTGCCGTTCAAATAGGGCGGCACCTTGACGGTACCTAACCAGAAAGCCACGGCTAACTACGTGCCAGCAGCCGCGGTAATACGTAGGTGGCAAGCGTTGTCCGGAATTATTGGGCGTAAAGGGCTCGCAGGCGGTTTCTTAAGTCTGATGTGAAAGCCCCCGGCTCAACCGGGGAGGGTCATTGGAAACTGGGGAACTTGAGTGCAGAAGAGGAGAGTGGAATTCCACGTGTAGCGGTGAAATGCGTAGAGATGTGGAGGAACACCAGTGGCGAAGGCGACTCTCTGGTCTGTAACTGACGCTGAGGAGCGAAAGCGTGGGGAGCGAACAGGATTAGATACCCTGGTAGTCCACGCCGTAAACGATGAGTGCTAAGTGTTAGGGGGTTTCCGCCCCTTAGTGCTGCAGCTAACGCATTAAGCACTCCGCCTGGGGAGTACGGTCGCAAGACTGAAACTCAAAGGAATTGACGGGGGCCCGCACAAGCGGTGGAGCATGTGGTTTAATTCGAAGCAACGCGAAGAACCTTACCAGGTCTTGACATCCTCTGACAATCCTAGAGATAGGACGTCCCCTTCGGGGGCAGAGTGACAGGTGGTGCATGGTTGTCGTCAGCTCGTGTCGTGAGATGTTGGGTTAAGTCCCGCAACGAGCGCAACCCTTGATCTTAGTTGCCAGCATTCAGTTGGGCACTCTAAGGTGACTGCCGGTGACAAACCGGAGGAAGGTGGGGATGACGTCAAATCATCATGCCCCTTATGACCTGGGCTACACACGTGCTACAATGGGCAGAACAAAGGGCAGCGAAACCGCGAGGTTAAGCCAATCCCACAAATCTGTTCTCAGTTCGGATCGCAGTCTGCAACTCGACTGCGTGAAGCTGGAATCGCTAGTAATCGCGGATCAGCATGCCGCGGTGAATACGTTCCCGGGCCTTGTACACACCGCCCGTCACACCACGAGAGTTTGTAACACCCGAAGTCGGTGAGGTAACCTTTTTGGAGCCAGCCGCCGAAGTGA |

**Table S2.** Volatile compounds content and aroma characteristics of *B. amyloliquefaciens* ZZ7.

| Number | Name | CAS | Content（μg/L） | Aroma characteristics | |
| --- | --- | --- | --- | --- | --- |
| 1 | 2,3-Butanediol | 513-85-9 | 274.99 | | cream and butter |
| 2 | Ethyl phenylacetate | 101-97-3 | 6.15 | | rose and honey |
| 3 | Trimethyl-pyrazine | 14667-55-1 | 0.78 | | nutty |

**Table S3.** Enzymes involved in metabolic pathways of sulfur compounds and their chemical reactions in *B. amyloliquefaciens* ZZ7.

| Enzyme | Chemical reaction | Pathway |
| --- | --- | --- |
| Sulfate adenylyltransferase | ATP + sulfate = diphosphate + adenylyl sulfate | Sulfate reduction |
| Adenylyl-sulfate kinase | ATP + adenylyl sulfate = ADP + 3'-phosphoadenylyl sulfate | Sulfate reduction |
| 3'(2'), 5'-Bisphosphate nucleotidase | adenosine 3',5'-bisphosphate + H2O = AMP + phosphate | Non-pathway related |
| Alkanesulfonate monooxygenase | an alkanesulfonate + FMNH2 + O2 = an aldehyde + FMN + sulfite + H2O | Non-pathway related |
| Phosphoadenylyl-sulfate reductase | adenosine 3',5'-bisphosphate + sulfite + thioredoxin disulfide = 3'-phosphoadenylyl sulfate + thioredoxin | Sulfate reduction |
| Methanesulfonate monooxygenase | methanesulfonate + FMNH2 + O2 = formaldehyde + FMN + sulfite + H2O | Non-pathway related |
| Assimilatory sulfite reductase | NH3 + 3 NADP+ + 2 H2O = nitrite + 3 NADPH + 5 H+ | Non-pathway related |
| Serine O-acetyltransferase | acetyl-CoA + L-serine = CoA + O-acetyl-L-serine | Cysteine metabolism |
| Cysteine synthase | L-cysteine + hydrogen cyanide = L-3-cyanoalanine + hydrogen sulfide | Cysteine metabolism |
| Homocysteine desulfhydrase | L-homocysteine + H2O = sulfide + NH3 + 2-oxobutanoate | Non-pathway related |
| Cystathionine γ-synthase | L-cystathionine + H2O = L-cysteine + 2-oxobutanoate + NH3 | Cysteine metabolism |
| Homoserine O-succinyltransferase | succinyl-CoA + L-homoserine = CoA + O-succinyl-L-homoserine | Methionine metabolism |
